# Investigation of the prevalence of latent tuberculosis in cancer patients compared to non-cancer patients: a case-control study

**DOI:** 10.3389/or.2024.1445678

**Published:** 2024-12-04

**Authors:** Masoud Mortezazadeh, Mehdi Karimi, Mohsen Esfandbod, Abbas Mofidi, Nima Hemmati, Mehdi Kashani, Niyousha Shirsalimi, Seyyed Taher Seyyed Mahmoudi, Ehsan Kamali Yazdi

**Affiliations:** ^1^ Department of Hematology-Oncology, Cancer Institute, Imam Khomeini Hospital Complex, Tehran University of Medical Sciences (TUMS), Tehran, Iran; ^2^ Faculty of Medicine, Bogomolets National Medical University (NMU), Kyiv, Ukraine; ^3^ Department of Hematology-Oncology, Sina Hospital, Tehran University of Medical Sciences (TUMS), Tehran, Iran; ^4^ Minimally Invasive Surgery Research Center (MISRC), Iran University of Medical Sciences (IUMS), Tehran, Iran; ^5^ Faculty of Medicine, Tehran University of Medical Sciences (TUMS), Tehran, Iran; ^6^ Faculty of Medicine, Hamadan University of Medical Science (UMSHA), Hamadan, Iran; ^7^ Faculty of Medicine, Tabriz University of Medical Sciences, Tabriz, Iran

**Keywords:** tuberculosis, infectious disease, cancer, tuberculin skin test, *Mycobacterium*, oncology

## Abstract

**Background:**

Latent tuberculosis (TB) can reactivate in immunocompromised individuals, such as cancer patients undergoing chemotherapy, leading to severe complications. Understanding the prevalence of latent TB in this high-risk group is crucial, especially in regions with moderate to high TB burdens.

**Aim:**

This study aims to determine the prevalence of latent tuberculosis in cancer patients before chemotherapy and immunotherapy to guide preventive interventions and reduce the risk of TB reactivation.

**Methods:**

This case-control study was conducted at Sina Hospital in Tehran, Iran, from 2012 to 2022. A total of 392, including 107 newly diagnosed cancer (case) and 285 non-cancer (control) patients, were enrolled in this study. All patients had received the *Bacillus* Calmette-Guérin (BCG) vaccine at the age of one. They underwent a thorough clinical examination and were screened using the tuberculin skin test (TST) to detect latent TB. Any active TB cases were identified through acid-fast smear tests. The data collected from the study participants was then analyzed.

**Results:**

The results showed no significant difference in the size of TST between cancer and non-cancer patients (cases: median = 2 mm, IQR: 1–12; controls: median = 2 mm, IQR: 1–5; *p* = 0.09). The prevalence of latent TB was 27.1% in cancer patients and 20.7% in non-cancer patients, with no significant association identified between latent TB and malignancies (P-value = 0.176). Over a median follow-up of 4 years, mortality was significantly higher in cancer patients compared to controls (42.1% vs 1.8%; P< 0.001, OR = 40.64). Additionally, deceased patients exhibited a greater prevalence of latent TB (44% vs 19.3% in survivors; P< 0.001, OR = 3.28), and increased size of TST was associated with higher mortality risk among cancer patients.

**Conclusion:**

In conclusion, this study emphasizes the need for vigilant latent TB screening in cancer patients, given the association between larger TST sizes and increased mortality risk. While no direct link between cancer type and latent TB was found, proactive TB management remains crucial, particularly for those undergoing immunosuppressive therapy.

## 1 Introduction

Tuberculosis (TB) is a complex infectious disease and a major global health concern, ranking among the leading causes of illness and death worldwide (1). In 2019, the World Health Organization (WHO) reported that around 10 million people were affected by TB, with 1.4 million dying from the disease. Additionally, a quarter of the world population was infected with *Mycobacterium tuberculosis* (MTB). TB is predicted to affect more than 25% of the global population (2). Its epidemiology varies significantly across different regions and populations, influenced by factors such as racial/ethnic background, geographical location, and socioeconomic conditions (3). Iran is an endemic region for TB, which remains a significant public health challenge due to high prevalence rates, regional disparities in drug resistance, and rising multidrug-resistant TB (MDR-TB) cases (4).

Most people with *Mycobacterium* TB infection have an early host defense that prevents the spread of disease, the infection remains latent or is eradicated, and the person is asymptomatic and noninfectious (5). Available tests for immune responses to TB antigens include the Tuberculin Skin Test (TST) and the interferon-gamma release assay (IGRA). Neither IGRA nor TST have a definite advantage in forecasting the likelihood of future active TB. A test should be chosen based on criteria, cost, and availability. False-negative TST results may occur in the setting of very recent infection or immunosuppression (6).

For most, LTBI remains dormant without progressing to active TB; however, individuals with compromised immune systems (e.g., autoimmune disease, HIV infection, cancer) are at heightened risk of reactivation (7, 8). Cancer patients, particularly those undergoing immunotherapy or chemotherapy, represent a vulnerable group due to their immunosuppressed state, which can facilitate the transition from latent to active TB (9, 10). Thus, understanding the prevalence of LTBI among cancer patients prior to chemotherapy is crucial for developing preventive strategies, reducing complications, and improving patient outcomes.

Research on latent TB in cancer patients is limited, with unclear data on whether they face higher risks or if specific cancer types affect LTBI prevalence. The impact of latent TB on prognosis and mortality in cancer patients also remains uncertain. Improved understanding of LTBI prevalence and outcomes could help clinicians implement preventive measures, benefiting TB management and cancer care. This study aims to assess LTBI prevalence in cancer patients, investigate its association with specific cancer types, and evaluate its impact on prognosis and mortality.

## 2 Materials and methods

### 2.1 Study design and sitting

This retrospective case-control study was conducted at *Sina Hospital* in Tehran, Iran, over a 10-year period from January 2012 to September 2022. The study aimed to assess the prevalence of latent TB infection in newly diagnosed cancer patients before chemotherapy and immunotherapy.

### 2.2 Participants (Eligible criteria)

A total of 392 adult patients with suspected cancer were initially included in the study. Following pathology-confirmed diagnoses, 107 patients with confirmed cancer were assigned to the case group, while the remaining 285 non-cancer patients were assigned to the control group. All participants had received *Bacillus* Calmette-Guérin (BCG) vaccination within the first year of life to account for baseline immunity.

To minimize confounding factors, participants were excluded if they met any of the following criteria: active TB infection, previous chemotherapy or immunotherapy, current or prior immunosuppressive therapy, delayed BCG vaccination, pregnancy, HIV infection, history of organ transplantation, autoimmune diseases, prior malignancies, recent surgeries, liver or kidney disease, or substance abuse. Patients with these conditions were excluded to prevent potential interference with the study outcomes and to ensure comparability between the case and control groups.

### 2.3 Examination of patients and latent TB screening

Patient demographic data and history of *Bacillus* Calmette-Guérin (BCG) vaccination were collected in detail through patient interviews and medical records. All participants were screened for latent LTBI using the TST. For the TST, 0.1 mL of purified protein derivative (PPD) was injected intradermally into the inner forearm using a tuberculin syringe with the needle bevel upward. The test results were read 48–72 h post-injection, with induration measured in millimeters across the forearm, perpendicular to the long axis. If the initial injection did not produce a 6–10 mm wheal or if the patient did not return within 72 h, the TST was repeated. Follow-up continued over approximately 4 years to monitor mortality and disease prognosis among the participants.

### 2.4 Assessment of active tuberculosis

All patients underwent comprehensive examinations to identify any signs of active TB, particularly pulmonary symptoms (e.g., persistent cough, sputum production, fever, chills, weight loss, night sweats, pleuritic chest pain) and extrapulmonary symptoms indicative of TB. If clinical suspicion for active TB arose from patient history, physical examination, or chest X-ray findings, further diagnostic tests were conducted. This included the IGRA and collection of pharyngeal samples for acid-fast smear and culture testing. Patients testing positive on the acid-fast smear or TB culture were excluded from the study to focus exclusively on latent TB cases.

This meticulous screening and exclusion process, coupled with a rigorous assessment of patient history, ensured a robust foundation for investigating the prevalence and clinical implications of latent TB in newly diagnosed cancer cases, as well as its potential impact on patient prognosis.

### 2.5 Ethical consideration

This study was approved by the Ethics Committee of Sina Hospital, Tehran University of Medical Sciences (TUMS), under code IR. TUMS.SINAHOSPITAL.REC.1399.068. Informed consent was obtained from all participants, who were assured of confidentiality and their right to withdraw at any time. All data was anonymized and securely stored to protect participant privacy.

### 2.6 Statistical analysis

This study utilized various statistical methods to analyze the relationships between latent TB, malignancies, and mortality. T-tests and chi-square tests were employed to compare demographic and clinical characteristics between cases and controls. To investigate the association between latent TB and malignancies, linear and logistic regression analyses were performed, adjusting for age and sex. Descriptive statistics, including the Mann-Whitney U and chi-square tests, were used to compare median TST sizes and latent TB frequencies between cases and controls. Both unadjusted and adjusted linear and logistic regression models assessed the relationship between latent TB and malignancies. Logistic regression and chi-square tests were used to evaluate mortality differences, identifying significant predictors such as age and TST size.

## 3 Results

### 3.1 Patients characteristics

**FIGURE 1:**
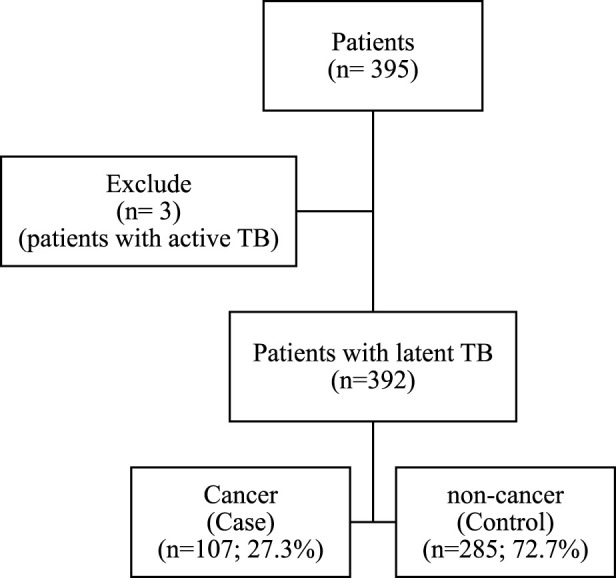
Flowchart of study.

Initially, 395 patients were included in the study. After excluding three patients due to active TB, 392 participants remained, with 107 (27.3%) classified as cancer patients in the case group and 285 (72.7%) as non-cancer patients in the control group, resulting in a control-to-case ratio of 2.6:1 (Figure 1).

Gender distribution was similar between groups, with 41 (38.3%) males and 66 (61.7%) females in the cancer group, compared to 120 (42.1%) males and 165 (57.9%) females in the non-cancer group. The difference in gender distribution between the two groups was not statistically significant (*p* = 0.479). However, a significant difference was observed in age, with the mean age of cancer patients being 53.7 years (±11.81) compared to 37.71 years (±12.49) for non-cancer patients (*p* = 0.001) (Table 1).

**TABLE 1:** Frequencies and demographic data of patients.

		Total	Cancer	Non-cancer	P-value
Frequency		392 (100%)	107 (27.3%)	285 (72.7%)	-
Gender	Male	161 (41.1%)	41 (38.3%)	120 (42.1%)	0.479
	Female	231 (58.9%)	66 (61.7%)	165 (57.9%)	
Age	year	42.08 ± 14.21	53.7 ± 11.81	37.71 ± 12.49	0.001

^a^ mean standard deviation (SD).

### 3.2 Types of cancers and latent TB

Among the 107 cancer patients in the study, the most common type of malignancy was breast cancer, affecting 40 individuals (37.4%), followed closely by gastrointestinal (GI) cancer, with 36 cases (33.6%). Lymphoma was the third most frequent, seen in 10 patients (9.3%). Other cancers included urogenital cancers in 7 patients (6.5%), gynecologic cancers in 6 patients (5.6%), hepatobiliary and lung cancers, each affecting three patients (2.8%), and head and neck cancers in 2 patients (1.9%). This distribution shows a predominance of breast and GI cancers within the cancer patient group (Table 2).

**TABLE 2:** Frequency of different malignancies in cancer patients (case group) (n = 107).

Malignancies	Cancer patients (n = 107)
Breast cancer	40 (37.4%)
Gastrointestinal cancer	36 (33.6%)
Lymphoma cancer	10 (9.3%)
Urogenital cancers	7 (6.5%)
Gynecologic cancer	6 (5.6%)
Hepatobiliary cancer	3 (2.8%)
Lung cancer	3 (2.8%)
Head and neck cancers	2 (1.9%)

There were no associations between having latent TB and having malignancies when latent TB was considered as an independent variable both in linear (unadjusted: B = 0.912, *p*-value = 0.301; adjusted: B = 0.750, *p*-value = 0.466) and logistic regression (unadjusted: OR = 1.42, 95%CI 0.85–2.38, *p*-value = 0.177, adjusted: OR = 1.34, 95%CI 0.73–2.47, *p*-value = 0.377) analysis with and without adjustment for age and sex.

The patients with GI cancer, in comparison to other types of cancer, had twice the chance of latent TB with an odds ratio of 2.1. The patients with urogenital cancer, in comparison to other types of cancer, had twice the chance of latent TB with an odds ratio of 2.8. However, when they adjusted for age, the results were not significant.

### 3.3 TST size and latent TB

**FIGURE 2:**
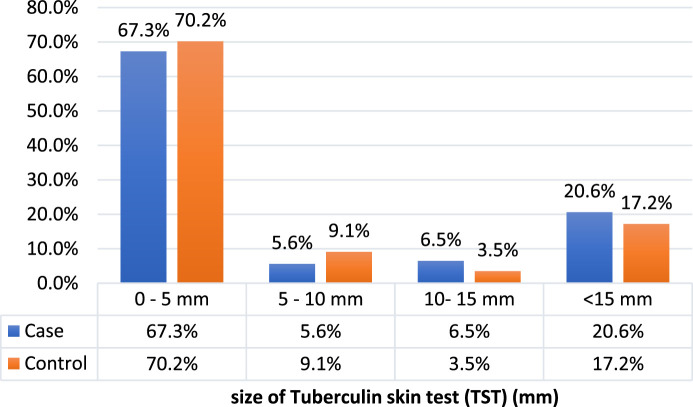
Size of TST in cancer (case) and non-cancer (control) group patients.

The median size of TST in millimeters did not differ in the case and control groups (cases: median = 2, IQR: 1,12, controls: median = 2, IQR: 1,5, P-value = 0.090). The frequency of latent TB was 27.1% (n = 29) in the case group and 20.7% (n = 59) in the control group (*p*-value = 0.176) (Figure 2; Table 3).

**TABLE 3:** Size of TST and frequency of latent TB in case and control groups.

	Case	Control	P-value
TST size (median; IQR)	2 (1–12)	2 (1–5)	0.090
Latent TB (n; %)	29 (27.1%)	59 (20.7%)	0.176

Tuberculin skin test (TST); Interquartile range ((IQR).

### 3.4 Mortality

**FIGURE 3:**
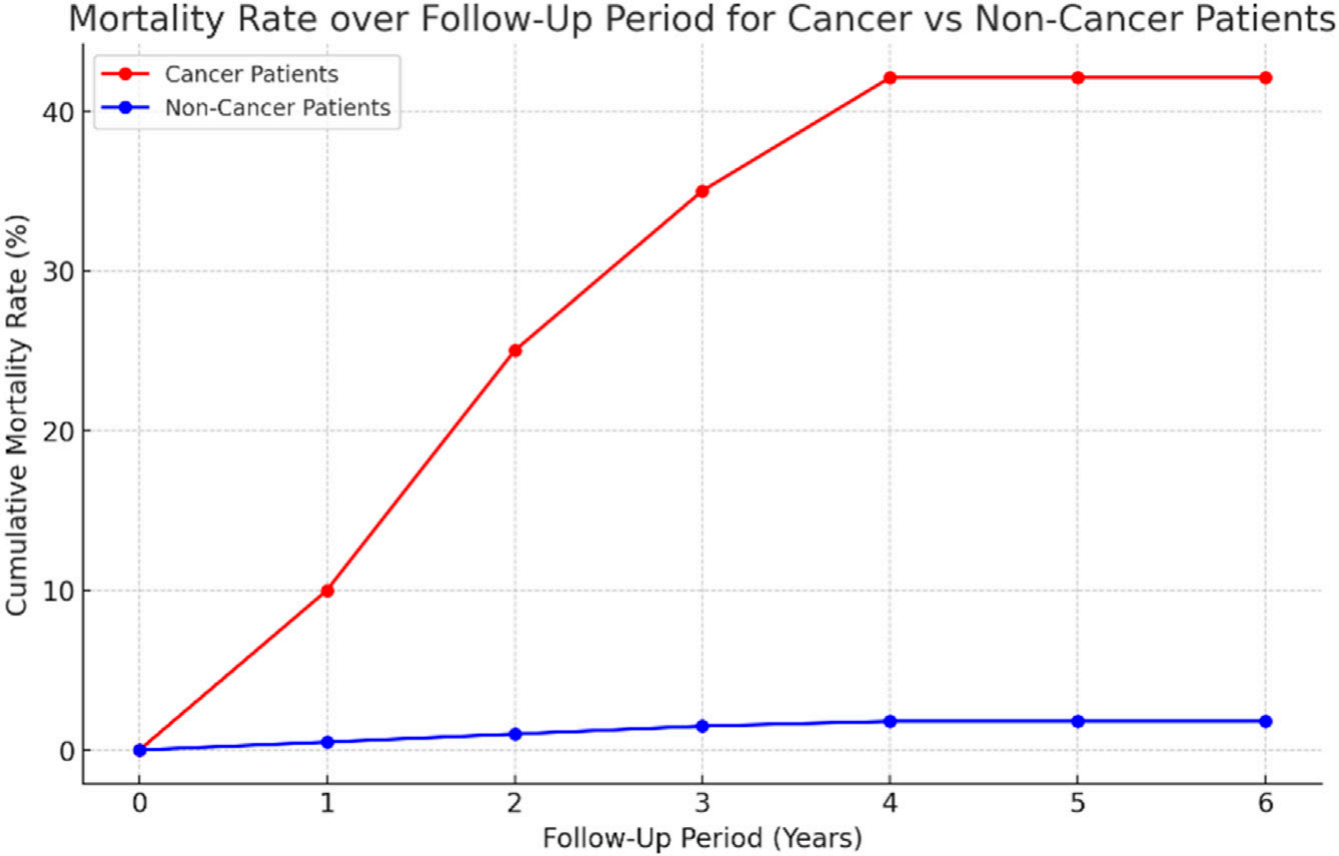
Line graph of the cumulative mortality rates over a follow-up period of 6 years for cancer and non-cancer patients. The graph highlights the significantly higher mortality among cancer patients (reaching 42.1%) compared to non-cancer patients (1.8%).

The median follow-up period was 4 years (IQR 2,6); generally, 12.8% (50) of patients passed away. There was a significant difference in mortality between the cases and controls through the follow-up period. The results revealed that the cancer patients had much higher odds of mortality in comparison to the non-cancer patients (42.1% [n = 45] vs 1.8% [n = 5], *p*-value <0.001, OR = 40.64, 95%CI, 15.5–106.58) (Figure 3).

The mean age of the surviving patients was 40.28 ± 13.47 versus 54.38 ± 13.1 in deceased (*p*-value <0.001). However, there was no significant difference between ages regarding mortality (female: survived 60.2% versus decreased 50.0%, *p*-value = 0.169). The size of TST was significantly higher in deceased patients (median = 5, IQR 2–20 versus median = 2, IQR 1-5, *p*-value <0.001). The deceased patients had a much higher frequency of latent TB with a percentage of 44% (n = 22), and 19.3% (n = 66) of surviving patients were diagnosed with this infection (P-value <0.001, OR = 3.28, 95%CI 1.76–6.1).

The results of the Cox regression analysis indicated that both malignancies and an increase in size of TST in millimeters are associated with an increased mortality hazard (Table 4). Specifically, among cancer patients, the risk of death rises by 6% for each additional millimeter in size of TST, even after adjusting for age and sex.

**TABLE 4:** Results of COX regression analysis regarding the mortality of patients with and without malignancies and its relation to latent TB.

Variable	Hazard ratio	Lower CI	Higher CI	P-value
Size of TST (mm)	1.05	1.02	1.08	0.001
Malignancy	37.34	13.00	107.24	<0.001
Age	1.00	0.97	1.02	0.874
Male gender	1.46	0.804	2.66	0.213

^a^ Median; CI: confidence interval, mm: millimeters; HR: hazard ratio; Tuberculin skin test (TST).

## 4 Discussion

This study aimed to assess the prevalence of TB among cancer and non-cancer patients prior to chemotherapy and immunotherapy, as well as to explore the association between various malignancies and latent TB. The results revealed that breast cancer and GI cancer were the most common malignancies, but no significant link to latent TB was established; however, GI and urogenital cancers exhibited higher odds of latent TB (2.1 and 2.8, respectively), which lost significance after adjusting for age. Additionally, there were no significant differences in median TST sizes between cancer and control groups, with latent TB frequencies of 27.1% and 20.7%, respectively, indicating no substantial association with cancer status. Importantly, the study highlighted significantly higher mortality rates among cancer patients (42.1%) compared to non-cancer patients (1.8%). Notably, latent TB was more prevalent in deceased patients (44% vs 19.3%), and the analysis indicated that each additional millimeter increase in TST size was associated with a 6% increase in mortality risk, underscoring the potential impact of latent TB on patient outcomes in the oncology setting. These findings suggest a need for vigilant screening and management of latent TB in cancer patients, particularly those undergoing immunosuppressive treatments.

Our findings are consistent with studies indicating a higher TB prevalence among cancer patients. Al-Dabbagh et al. (11) found a higher TB prevalence among chemotherapy patients in Saudi Arabia, particularly in older patients and those with solid tumors. Similarly, Cheng et al. (12) found that individuals with hematologic, head, neck, and lung cancers have a ninefold higher risk of developing active TB compared to those without cancer. Osorio-López et al. (13) reported a 31.2% prevalence of latent TB in patients with hematological neoplasms, noting good tolerance and adherence to isoniazid treatment. In contrast, Malek et al. (14) demonstrated that while latent TB reactivation is rare in cancer patients treated with checkpoint inhibitor immunotherapy, hepatotoxicity is common, necessitating close monitoring to prevent hepatic injury and disruption of latent TB therapy. A study by Ganzel et al. (15) found that patients with hematological malignancies, particularly lymphoma, have a higher incidence of TB after cancer diagnosis, but Hodgkin lymphoma patients do not have a higher risk compared to other hematological malignancies. Bourlon et al. (16) latent TB is prevalent among hematopoietic stem cell transplant recipients and donors, increasing the risk of active TB post-transplantation and higher risk of future cancers, including multiple myeloma, lung, kidney, bladder, hepatobiliary, and GI.

Numerous studies support our findings, indicating that cancer patients have a higher risk of developing active TB from latent TB, which is associated with increased mortality rates. Mishra et al. (17) found that cancer patients with latent TB are at a higher risk of developing active TB due to their immunocompromised state from cancer or treatment methods like chemo-radiotherapy, emphasizing the need for vigilant screening and preventive strategies. Fan et al. (18) found that 25% of newly diagnosed lung cancer patients had latent TB, highlighting the considerable risk of concurrent latent TB in these patients. This finding aligns with our results, emphasizing the necessity for routine latent TB screening in newly diagnosed cancer patients to mitigate the risk of TB activation (19). Abdelwahab et al. (20) identified several risk factors for death from TB in cancer patients, including lung cancer, presence of metastasis, and poor performance status (ECOG ≥3). Interestingly, previous chemotherapy treatment was found to be a protective factor against mortality from TB in cancer patients, suggesting that specific treatment regimens might confer some level of protection against TB mortality. This protective effect might be due to the immunomodulatory effects of chemotherapy, which could potentially influence the progression of latent TB to active TB (20).

Older age is a significant risk factor for latent TB among cancer patients. For instance, a study found that the mean age was significantly higher in patients with positive compared to those with negative results (11). Several studies indicated that certain types of cancer are more associated with latent TB. For example, Aldabbagh et al. (11) reported that 92% of patients with positive IGRA had solid cancers, and all active TB cases occurred in this group. A study in Taiwan found that non-adenocarcinoma lung cancer patients had a higher proportion of latent TB compared to those with adenocarcinoma, especially in younger patients (21). Another study highlighted that lung cancer patients receiving immune checkpoint inhibitors (ICIs) had a higher incidence of TB compared to those receiving tyrosine kinase inhibitors (TKIs) (9).

A recent study conducted by Chen et al. (9) showed that cancer patients receiving immunosuppressive medication, like chemotherapy or immune checkpoint inhibitors (ICIs), are more likely to experience latent TB reactivation, with ICIs being particularly linked to increased TB reactivation. WJ Su et al. (21) found that Chronic Obstructive Pulmonary Disease (COPD) is an independent predictor of latent TB in Taiwanese lung cancer patients, and fibrocalcified lesions on chest radiograms were also significant predictors. Research has indicated that male cancer patients are more susceptible to contracting tuberculosis (22). Foreign birth or having foreign-born parents has been discovered as a risk factor for TB among children with cancer and those who have had bone marrow transplantation (23).

Considering these risk factors, it is imperative to establish screening protocols for latent TB in cancer patients, particularly those with solid tumors, advanced age, and those receiving immunosuppressive treatments. Timely identification and management of latent TB can effectively hinder the development of active tuberculosis, a crucial consideration for cancer patients with impaired immune systems (9, 11, 22, 23). Active latent TB screening and treatment at the outset of cancer therapy may help minimize the reactivation risk of active TB illness and related mortality in cancer patients (17).

### 4.1 Strength and limitations

This study’s strengths include a comprehensive screening protocol and detailed demographic data, which help improve understanding of participants’ health profiles. Rigorous exclusion criteria reduced confounding factors, and a 4-year follow-up period allowed for valuable insights into the long-term outcomes of newly diagnosed cancer patients before treatment. The use of various statistical analyses further strengthened the reliability of the findings, and the study’s setting in a TB-endemic region like Iran provides practical insights into the complexities of latent TB management in vulnerable populations. However, the study’s retrospective, single-center design limits the generalizability of results, and reliance on the TST rather than the more accurate IGRA test due to financial and regional limitations is a significant drawback. Selection bias may have occurred due to strict inclusion criteria, potentially leaving some confounding factors unaddressed. Additionally, the small sample size of the cancer group limits statistical power, and changes in TB prevalence and cancer treatments over the decade-long study period may reduce the relevance of the findings to current clinical contexts.

## 5 Conclusion

This study highlights the importance of proactive TB screening and management in cancer patients, particularly given the elevated mortality rate associated with latent TB in this population. While no significant association was found between specific cancer types and latent TB, the observed increase in mortality risk with larger TST sizes suggests that latent TB may negatively impact outcomes in oncology settings. These findings support vigilant TB monitoring as a critical component of care for cancer patients, especially those receiving immunosuppressive treatments. Future research should focus on developing tailored screening and preventive strategies to better understand and mitigate the risks of latent TB in cancer patients.

## Data Availability

The original contributions presented in the study are included in the article/Supplementary Material, further inquiries can be directed to the corresponding authors.

## Abbreviations

TB, Tuberculosis; MTB, *Mycobacterium tuberculosis*; TST, Tuberculin skin test; PPD, Purified protein derivative; BCG, *Bacillus* Calmette-Guérin; IGRA, Interferon-gamma release assay; GI, Gastrointestinal.
